# Child mortality in the Democratic Republic of Congo: cross-sectional evidence of the effect of geographic location and prolonged conflict from a national household survey

**DOI:** 10.1186/1471-2458-14-266

**Published:** 2014-03-20

**Authors:** Ngianga-Bakwin Kandala, Tumwaka P Mandungu, Kisumbula Mbela, Kikhela PD Nzita, Banza B Kalambayi, Kalambayi P Kayembe, Jacques B O Emina

**Affiliations:** 1 Division of Health Sciences, Populations, Evidence and Technologies Group, Medical School Building, The University of Warwick, Warwick Medical School, Coventry CV4 7AL, UK; 2 KEMRI-University of Oxford-Wellcome Trust Collaborative Programme, Malaria Public Health and Epidemiology Group, Centre for Geographic Medicine, University of Oxford, Nairobi, Kenya; 3 Division of Epidemiology and Biostatistics, School of Public Health, University of Witwatersrand, Johannesburg, South Africa; 4 Institut National de Statistique, Ministère du Plan, Kinshasa, Democratic Republic of Congo; 5 Département des Sciences de la Population et du Développement, Faculté des Sciences Economiques, Université de Kinshasa, B.P. 176, Kinshasa XI, Democratic Republic of Congo; 6 School of Medicine, University of Kinshasa, B.P. 1580, Kinshasa, Democratic Republic of Congo; 7 INDEPTH Network, Accra, Ghana

**Keywords:** U5M, Conflict, Millennium development goals, Geographic patterns, DRC

## Abstract

**Background:**

The child mortality rate is a good indicator of development. High levels of infectious diseases and high child mortality make the Democratic Republic of Congo (DRC) one of the most challenging environments for health development in Sub-Saharan Africa (SSA). Recent conflicts in the eastern part of the country and bad governance have compounded the problem. This study aimed to examine province-level geographic variation in under-five mortality (U5M), accounting for individual- and household-level risk factors including environmental factors such as conflict.

**Methods:**

Our analysis used the nationally representative cross-sectional household sample of 8,992 children under five in the 2007 DRC Demographic and Health Survey. In the survey year, 1,005 deaths among this group were observed. Information on U5M was aggregated to the 11 provinces, and a Bayesian geo-additive discrete-time survival mixed model was used to map the geographic distribution of under-five mortality rates (U5MRs) at the province level, accounting for observable and unobservable risk factors.

**Results:**

The overall U5MR was 159 per 1,000 live births. Significant associations with risk of U5M were found for < 24 month birth interval [posterior odds ratio and 95% credible region: 1.14 (1.04, 1.26)], home birth [1.13 (1.01, 1.27)] and living with a single mother [1.16 (1.03, 1.33)]. Striking variation was also noted in the risk of U5M by province of residence, with the highest risk in Kasaï-Oriental, a non-conflict area of the DRC, and the lowest in the conflict area of North Kivu.

**Conclusion:**

This study reveals clear geographic patterns in rates of U5M in the DRC and shows the potential role of individual child, household and environmental factors, which are unexplained by the ongoing conflict. The displacement of mothers to safer areas may explain the lower U5MR observed at the epicentre of the conflict in North Kivu, compared with rates in conflict-free areas. Overall, the U5M maps point to a lack of progress towards the Millennium Development Goal of reducing U5M by half by 2015.

## Background

The child mortality rate is considered the best proxy indicator of general population health and the level of socioeconomic development [1]. The child mortality rate is also a useful marker of overall development and a Millennium Development Goal (MDG) indicator [2]. A high rate of mortality among children reflects precarious conditions such as poor nutrition, low access to drinking water and inadequate health services [1]. In Sub-Saharan Africa (SSA), several conditions influence infant mortality, including hygienic, socioeconomic, cultural, environmental and geographic factors [3]. However, geographical associations with mortality have been neglected. Thus, it is a worthwhile endeavour to investigate the trends, geographic patterns and associations of child mortality rates [4].

**Figure 1:**
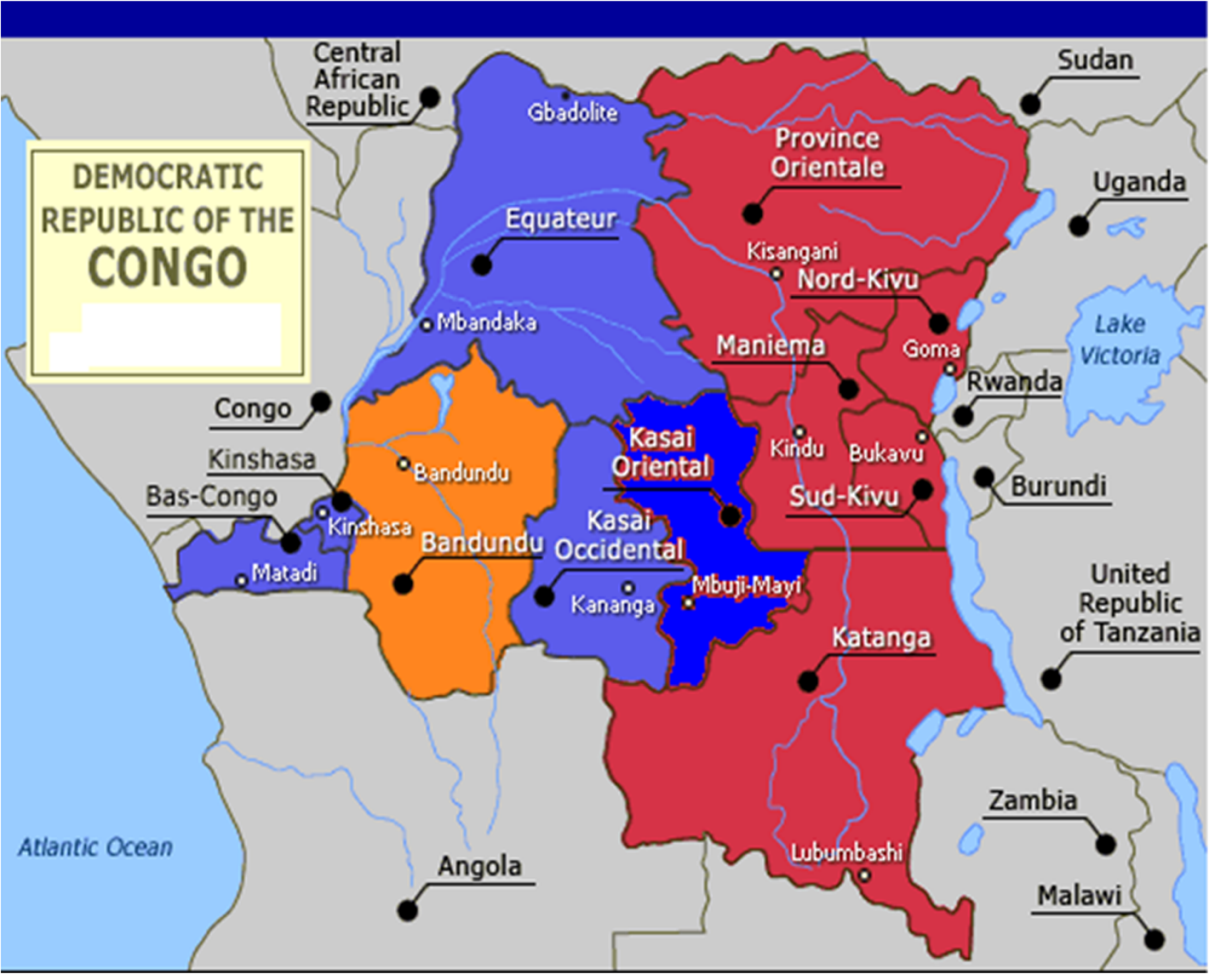
**Map of the Democratic Republic of Congo showing crude under-five mortality rate by province.** Overall mortality rate was 148 deaths per 1000 live births (weighted data). Provinces in red are conflict-affected areas Kinshasa: 31 deaths per 1000 live births, Bas-Congo: 66, Bandundu: 57, Équateur: 74, Orientale: 100, North Kivu: 47, South Kivu: 68, Maniema: 103, Katanga: 66, Kasaï-Oriental: 68, Kasaï Occidental: 69.

The DRC is one of the most challenging environments for health development in SSA. Of SSA countries, the DRC has the third largest population and the second largest land area, distributed across 11 provinces (see Figure 1). The DRC has high rates of infectious disease and child mortality [5-7]. One reason for this is the country’s reliance upon a physical and health infrastructure that has suffered from a lack of investment and fallen prey to decades of protracted conflict, poor governance and economic mismanagement [8-12]. A second factor involves the uneven distribution of access to health care, health services infrastructure and development. Urban areas and provinces such as Bas-Congo, Katanga and the capital city, Kinshasa, perform fairly well, but rural areas and provinces such as Bandundu, Kasaï-Oriental, Kasaï-Occidental, Maniema, Équateur, North Kivu and South Kivu lack the health infrastructure adequate to address child mortality issues [10-12]. A third factor is that the recent conflict that has exacerbated this situation [13-15]. The known predictors of mortality are generally linked with food security and accessibility, especially in conflict areas such as the provinces of Orientale, Maniema, Katanga, North Kivu, South Kivu and, more recently, Équateur [11,13].

The often disastrous impact of complex emergencies in the DRC on public health has been widely documented by international entities and nongovernmental organisations [12-16]. Since 1996, the DRC has been hit by a conflict known as “Africa’s first world war”, involving at least six nations in the region. The war has devastated and destabilised the country, claiming the lives of about six million civilians [13-16]. Compared with other conflicts over the past centuries, the consequences have been similar in nature but on a much greater scale [8]. Despite the presence of more than 17,000 United Nations (UN) peacekeepers, a major UN deployment, the situation in the DRC continues to be a matter of great concern for the international community [14-16]. The combined effects of this war and the preceding decades of poor governance and mismanagement have contributed to the impoverishment of the DRC [8-12].

DRC’s estimated population of nearly 70 million people live in abject poverty [5]. In the health sector, the low level of social indicators shows the catastrophic impact that the conflict has had on living conditions, particularly for women and children. Life expectancy at birth, which was estimated at 52.4 years in 1994, fell to 45 years or less by 2004 [5,13,17-20]. In many parts of the country, people live in dismal conditions, and what remains of foreign armies in the DRC continue to cause havoc and adversely affect public health services [13]. Various militia groups operating in the eastern provinces continue to use rape as an instrument of war and destroy an already inadequate public health service [12-15].

Despite the vast mineral wealth of the country, health, nutrition and population outcomes in the DRC remain extremely poor. This makes it difficult for the country to achieve the MDGs. In fact, the United Nations Human Development Report 2011 ranked the DRC as the last country in the world (187 out of 187 countries) in terms of the Human Development Index [21,22]. Over one-third of children under age five in the DRC are chronically malnourished (stunting), and 16% suffer from acute malnutrition (wasting), reflecting a wide vulnerability to short-term crises. The infant mortality rate is above the overall average for Africa of 126 per 1,000 and stands out as one of the most alarming in the region [17-20,23,24]. Crude national under-five mortality rates (U5MRs) from various household surveys have declined slightly in the past decades from 220 per 1,000 live births in 1995 (MICS) to 213 in 2001 (MICS2) and 148 in 2007 (DHS) [5,23,24]. However, the available figures suggest that child mortality in the DRC remains among the highest in the world [1,17-20,23,24]. The high U5MRs and the last-place ranking on human development indicators suggest that little progress has been made in the implementation of the government’s Priority Action Plan on the National Acceleration Framework to reduce infant and maternal mortality in the DRC [17-19,21-25].

In this context, our study used data from the 2007 DRC Demographic and Health Survey (DRC-DHS) to investigate under-five mortality (U5M) at the provincial level. To produce robust estimates, we adopted novel approaches to take into account uncertainty in sampling variation given the existence of various measured and unmeasured factors such as conflicts. The findings of this study highlight geographic patterns that may provide insights for humanitarian intervention and policy formulation.

## Methods

### Data

For the 2007 DRC-DHS, data were collected from a nationally representative sample of 9,000 households (3,690 in urban areas and 5,310 in rural areas). Complete interviews were conducted with 9,995 women aged 15–49 and 4,757 men aged 15–59 [5]. In addition to standard modules or sets of questions, we sought to establish background characteristics such as contraceptive knowledge and practice, marriage and AIDS knowledge, complete birth history, nutrition, immunisation and health information about each child under 5 years of age at the time of the survey.

Birth history data were collected for each of the women interviewed. For each birth, questions were asked about the date of birth, name, sex, survival status and age at death if deceased. This information allowed us to investigate mortality patterns and unearth the determinants of U5M during this period. This study uses the available information on 9,995 women and 8,992 live births occurring in the 5 years preceding the survey. In general, the DHS data are of good quality, covering all regions including both urban and rural areas in the DRC [5]. However, the survey is cross-sectional. The 2007 DHS was carried out after the 2006 elections (from February 2 to April 30, 2007, for Kinshasa and from May 10 to August 31, 2007, for the remaining provinces). At the time, some villages and municipalities in the eastern provinces of North Kivu, South Kivu and Orientale were still experiencing armed conflict [5]. Therefore, the results of our study might be affected by data quality issues owing to undercoverage or bias because of nonresponse or temporary migration caused by conflict. Ethical approval for this project was granted by the Ethics Committee of the DRC Ministry of Planning and Macro International [5].

### Statistical analysis

Data were analysed with relevant variables to carry out survival analyses for the first 5 years of life (U5M: 5q0). The response variable was defined as y_i_ = 1 if a child died in the 5 years preceding the survey and y_i_ = 0 otherwise. The standard measure of effect was the odds ratio (OR) and the 95% credible region (CR). We examined spatial variation in U5M with a flexible Bayesian geo-additive discrete-time survival model. This models mortality events as person-specific Cox processes while controlling spatial dependence and possibly nonlinear effects of covariates within a simultaneous and coherent regression framework [26-28]. The analysis was carried out using version 2.9 of the BayesX software package [29], which permits Bayesian inference based on Markov chain Monte Carlo (MCMC) simulation techniques. The statistical methods used have been discussed elsewhere [4,27-34] and are also provided as an additional file (see Additional file 1).

## Results

In the 5-year period preceding the survey, there were 1,005 reported deaths. Baseline characteristics of the study population are displayed in Table 1 for the overall sample of births in the last 5 years (N = 8,992). For comparison purposes, Tables 2 and 3 display recent estimates of U5MRs in Central Africa and longer term U5M trends in the DRC. Overall, the U5MR (defined as the probability of dying between birth and exactly 5 years of age, expressed per 1,000 live births) was 159 (152.9, 164.9) per 1,000 live births (un-weighted data). U5MRs were higher in rural areas compared with urban areas (184.1 vs. 120.4), and U5MR was higher among males than females (168.2 vs. 149.2). On average, U5MR was also higher among children with a short preceding birth interval (< 24 months) and among children born to older women, those in low-income households, those with low maternal education and those with unmarried mothers (Table 1).

**Table 1:** Under-five mortality rates and 95% confidence intervals by background characteristics [un-weighted data] (DRC-DHS, 2007)

**Background characteristics**	**U5M rates**	**95% CI**	
***Type of place of residence***		**Low**	**Upper**
Urban	120.4	112.2	129.2
Rural	184.1	176.1	192.5
*Sex*			
Male	168.2	159.8	177.1
Female	149.2	141.1	157.7
*Preceding birth interval*			
< 24 months	214.3	200.9	228.4
≥ 24 months	133.5	126.1	141.2
*Maternal age at birth*			
< 20 years	181.6	166.9	197.4
20–34	148.0	141.1	155.2
≥ 35	183.4	167.0	201.1
*Household wealth index*			
Low income household	191.4	180.5	202.9
Intermediate income household	167.3	157.1	178.2
High income household	116.4	107.6	125.9
*Maternal education*			
None	194.9	182.4	208.1
Primary	170.8	161.5	180.5
Secondary & higher	113.6	104.8	123.2
*Maternal marital status*			
Unmarried mother	170.5	152.4	190.6
Mother in union	157.4	151.2	163.8
*Province of residence*			
Kinshasa	94.3	80.7	110.0
Bas Congo	172.6	151.2	196.6
Bandundu	140.3	122.4	160.6
Équateur	162.0	144.3	181.8
Orientale	184.8	162.5	209.7
North Kivu	118.4	101.1	138.4
South Kivu	183.5	163.3	205.8
Maniema	207.8	187.0	230.5
Katanga	157.4	139.4	177.6
Kasaï-Oriental	162.9	145.1	182.7
Kasaï-Occidental	173.6	153.4	196.2
Overall	158.8	152.9	164.9

**Table 2:** Under-five mortality rates in Central Africa and the Great Lake countries

	**U5M**	**Low bound**	**Upper bound**
Angola	158	124	231
Burundi	139	116	199
Cameroon	127	107	135
Central African Republic	164	131	213
Chad	169	146	206
Congo Brazzaville	99	84	107
**DRC**	**168**	**139**	**235**
Equatorial Guinea	118	63	235
Gabon	66	50	81
Rwanda	54	47	67
Sudan	86	66	117
Tanzania	68	62	81
Uganda	90	84	105
Zambia	83	76	110

Source: UNICEF, WHO, The World Bank and United Nations (http://0nulled.com/doc/pdf/download/www__childinfo__org--files--Child_Mortality_Report_2012.pdf) retrieved on October 31, 2012.

**Table 3:** Trend in under-five mortality estimates in the DRC

	**U5MR**	**Source**
1984	213	1984 Census
1990	181	UNICEF, WHO, The World Bank and United Nations
1995	190	1995 MICS
2001	213	2001 MICS

Table 1 also shows the distribution of U5MRs by geographic location, revealing dramatic geographic disparities in observed crude U5MRs. The mortality rate is very high in the provinces of Maniema, Orientale and South Kivu, estimated at 207.8, 184.8 and 183.5 per 1,000 live births, respectively. Kinshasa and North Kivu are among the provinces with lower observed U5MRs compared with the national average.

**Figure 2:**
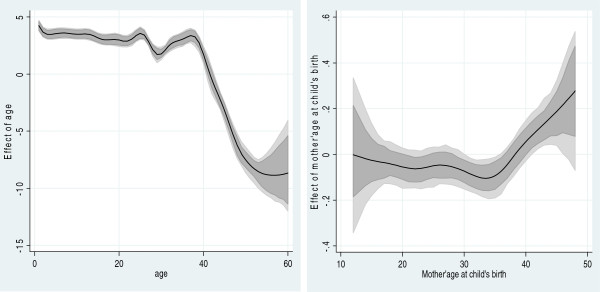
**Estimated nonparametric effects of baseline time child survival and mother’s age at child’s birth.** Left: Estimated nonparametric effect of baseline time child survival. The posterior mean within 80% credible regions is shown. Right: Estimated nonparametric effect of mother’s age at child’s birth. The posterior mean within 80% credible regions is shown.

Table 4 displays both marginal and posterior odds ratios of U5M risks across the selected study characteristics. Results from both standard logistic regression and multivariate Bayesian geo-additive survival analyses (right-hand column) provided evidence of the role of short birth interval, delivery at home and mother’s unmarried status as risk factors. Specifically, factors consistently associated with higher U5M included birth intervals of less than 2 years [posterior odds ratio and 95% credible region: 1.14 (1.04, 1.26)], home delivery [1.13 (1.01, 1.27], single status of mother [1.16 (1.03, 1.33] and living outside North Kivu. Urban residence, child’s gender, number of antenatal visits, mother’s education and mother’s wealth were not significantly associated with U5M risk. Nonlinear associations were identified between the baseline hazard of child survival and mother’s age at child’s birth as well as U5M using a flexible nonlinear curve (Figure 2).

**Table 4:** Unadjusted and fully adjusted odds ratios and 95% confidence intervals for the risk of under-five mortality by background characteristics (DRC-DHS, 2007)

**Variable**	**Unadjusted OR & 95% CI**	**Fully adjusted OR & 95% CI**
***National***		
***Type of place of residence***		
Urban	1.00	1.00
Rural	1.70 (1.47, 1.96)	1.09 (0.98, 1.25)
***Sex of child***		
Male	1.09 (0.96, 1.25)	1.03 (0.96, 1.13)
Female	1.00	1.00
***Preceding birth interval***		
< 24 months	1.65 (1.43, 1.91)	1.14 (1.04, 1.26)
≥ 24 months	1.00	1.00
***Mother’s age at child’s birth***		
≤ 20 years	1.14 (.98, 1.33)	See Figure 2
21–35 years	1.00	
***Antenatal visits***		
No antenatal visits	1.52 (1.15, 2.01)	1.01 (0.89, 1.14)
≥ 1 antenatal visit	1.00	1.00
***Place of delivery***		
Hospital	1.00	1.00
Home	1.66 (1.44, 1.91)	1.13 (1.01, 1.27)
***Asset index***		
Low income household	1.65 (1.41, 1.96)	1.04 (0.92, 1.16)
Middle income household	1.29 (1.06, 1.57)	1.03 (0.90, 1.18)
Higher income household	1.00	1.00
***Mother’s educational attainment***		
Up to primary	1.65 (1.42, 1.93)	1.01 (0.93, 1.12)
Secondary or higher	1.00	1.00
***Marital status of mother***		
Single	1.15 (0.93, 1.42)	1.16 (1.03, 1.33)
Married	1.00	1.00
***Province***		
Kinshasa	0.85 (0.59, 1.23)	0.98 (0.86, 1.08)
Bas Congo	1.37 (0.95, 1.97)	1.01 (0.92, 1.13)
Bandundu	1.36 (0.96, 1.92)	0.96 (0.87, 1.05)
Équateur	1.65 (1.18, 2.30)	0.99 (0.90, 1.10)
Orientale	1.59 (1.11, 2.27)	1.00 (0.89, 1.10)
North Kivu	1.00	0.92 (0.72, 1.02)
Maniema	1.89 (1.36, 2.63)	1.01 (0.92, 1.11)
South Kivu	1.58 (1.13, 2.23)	1.04 (0.94, 1.15)
Katanga	1.71 (1.23, 2.39)	1.02 (0.92, 1.13)
Kasaï-Oriental	1.76 (1.27, 2.44)	1.04 (0.95, 1.16)
Kasaï-Occidental	1.47 (1.04, 2.07)	1.01 (0.90, 1.11)

Unadjusted marginal odds ratios (OR) from standard logistic regression models. North Kivu province was used as the reference category because it had the lowest crude under-5 mortality (see Table 1).

Spatially adjusted posterior odds ratios (OR) from Bayesian geo-additive regression models after controlling for nonlinear effect of age, categorical variables and the province of residence (spatial effects).

Figure 2 shows the estimated baseline nonparametric hazard of child survival (left) and the effect of mother’s age at child’s birth (right). The main point of attention is the posterior means within the 80% credible regions. There is a pronounced effect of the baseline time on child survival during the first months of life (Figure 2 left), but the excess risk persists through the first 40-month period. The baseline effects peak at 24, 30 and 36 months. The nonlinear effect (U-shaped association) between mother’s age at child’s birth and mortality rate is clearly depicted in the right panel of Figure 2. Higher mortality rates are observed among mothers giving birth at older ages (38 years and above).

With regard to U5M risk in the marginal regression analyses, a striking variation was noted in the U5M risks across provinces. The highest risks were observed in Maniema province [1.89 (1.36, 2.63)], Kasaï-Oriental [1.76 (1.27, 2.44)] and Katanga [1.71 (1.23, 2.39)], whereas the lowest risks were found in North Kivu and Kinshasa [0.85 (0.59, 1.23)].

**Figure 3:**
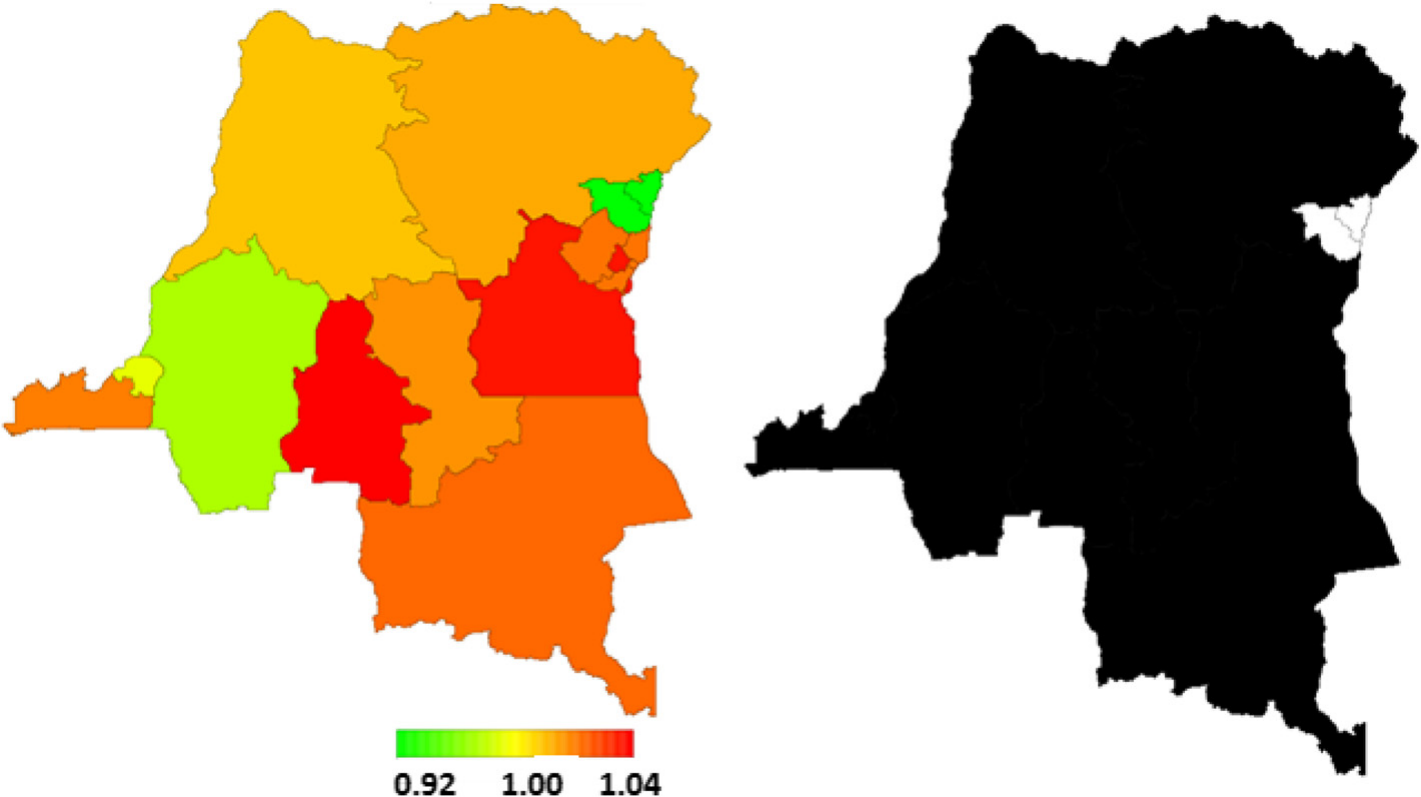
**Total residual spatial effects of child survival and corresponding posterior probabilities at 80% nominal level by province.** Left: Total residual spatial effects of child survival at province level in the DRC. Posterior odds ratio is shown. Right: Corresponding posterior probabilities at 80% nominal level; that is the level of confidence we have in the results, illustrated as a colour scale: white denotes provinces with strictly negative credible intervals (lower mortality), and black denotes provinces with strictly positive credible intervals (higher mortality).

Figure 3 shows the results for the covariate-adjusted spatial variation of U5M status captured in terms of the global effects across provinces (left) (i.e., the sum of local province effects and smoothed province effects). A clear pattern of provinces with higher risk of U5M was observed, mostly in the south-eastern province of Kasaï-Occidental and in the eastern provinces of South Kivu and Maniema. Provinces in the west and other provinces in the east, in contrast, were associated with a lower risk of U5M. These spatial patterns confirm the observed marginal model findings shown in Table 4.

Figure 3 (right) indicates the posterior probabilities at the 80% nominal level and illustrates the level of confidence we have in the results. This is illustrated as a colour scale, with white denoting provinces with strictly negative credible intervals (lower mortality) and black showing provinces with strictly positive credible intervals (higher mortality). The total residual spatial effects point to a slight advantage in terms of child survival in North Kivu. The survival advantage for children in Bas-Congo, Bandundu and Kinshasa is captured by the unstructured localised spatial effects (results not shown here), but this is statistically insignificant and shows how these local spatial effects are influenced by neighbouring effects.

## Discussion

The significance of this study lies in the finding that the high levels and variations of U5M in the DRC cannot be explained by the ongoing conflict. The study suggests that U5M is increasingly influenced by factors including hygienic and environmental conditions as well as socioeconomic and cultural variables, which persist within various social contexts [3]. Several risk factors beyond the well-typically considered variables were examined. The effect of geographic location was explored and quantified as a proxy for environmental factors such as conflict. The findings indicate that spatial effects mediate mortality rates in conflict-affected countries such as the DRC. The spatial location patterns of mortality mirror many unmeasured factors at the provincial level, such as unequal access to health services, exposure to conflict and the effect of public health policies on children’s health and survival.

In addition to the dramatic spatial inequalities observed in child mortality rates, the differential mortality observed from traditional risk factors (child’s sex, age of the mother and the interval between births) points to the important role of the geographic location where a child lives. The spatial patterns may also mirror factors such as high fertility, which is characterised by short birth intervals. Nationally, the U5M was estimated at 159 per 1,000 live births. This figure could be even higher in reality, because of the absence of official vital registries and because displacement and conflicts often influence record-keeping [12]. This level of child mortality in the DRC is unacceptably high, especially considering the achievability of rates below 10 deaths per 1,000 live births even in developing countries, U5M trends in the broader region as of 2007 (Table 2) and the DRC’s estimates prior to 1997, the year of the start of the ongoing conflict (Table 3) [19] .

The use of geo-additive modelling has been crucial to disentangle the role of various competing factors contributing to higher mortality in this context. At the time of the survey, some provinces were (and still are) affected by ongoing conflict (Maniema, North and South Kivu and Katanga). Although conflict undoubtedly confounds the observed mortality rates, this study shows that conflict is not the only factor contributing to the excess mortality risks. Possible explanatory factors are the lack of programmes to improve child health and survival and the lack of access to adequate health services. National programmes addressing this situation have since emerged (see, for example the Congolese Government’s National Committee for Health *A Promise Renewed* acceleration framework to reduce child mortality [http://blog.usaid.gov/2013/05/drc-making-great-strides-in-child-survival/]. However, in past decades, national programmes of this type have been lacking in implementation, leading to the position of the DRC as the lowest country worldwide in development indicators rankings [21,22].

This study points to the worrying state of child mortality in the DRC. Despite the pledge by the DRC Government to improve child health and survival by 2015, the child mortality rate remains very concerning in most parts of the country. Using weighted data, U5M has been estimated from the 2007 DRC Demographic and Health Survey (DRC-DHS) to be in the range of 148 to 159 per 1,000 live births [5]. These rates suggest that the DRC cannot attain the Millennium Development Goal to reduce U5M by half by 2015 (MDG 4) unless sustained efforts and investments are made in the entire country and not only in the most affected areas. To achieve MDG 4, the DRC needs to reduce its U5M by two-thirds, from the current 159 to 60 per 1,000 live births. Causes of the high child mortality rates include the precarious socioeconomic conditions and armed conflicts. The combined effect of these factors means that the DRC’s infrastructure is among the least adequate, worldwide, for supporting the health sector. Almost all sociodemographic indicators of the country are alarming [1,21,22].

The spatial effects in the present study also demonstrate provincial disparity in child health and survival as a consequence of precarious hygienic, social and economic conditions. There is a marked residual spatial effect of higher mortality risk in the provinces of Maniema and Katanga, where conflicts linger. However, even in non-conflict areas such as the Kasaï-Oriental province, the same alarming situation prevails. The lower rates of U5M observed in the provinces of Kinshasa and North Kivu are not surprising. Kinshasa is the capital city and maintains a minimally functioning health infrastructure and access to health services. In North Kivu, the epicentre of the ongoing conflict, several nongovernmental organisations have dedicated efforts and resources to reducing child mortality. This result is perhaps not surprising, because it mirrors the heavy humanitarian focus on this province and because most children live in camps because North Kivu is the epicentre of the conflict because of its valuable mineral resources (e.g., coltan and gold). However, another possible explanation for the finding of lower mortality in North Kivu is the inaccessibility of its entire population during the survey as a result of conflict. Indeed, the International Rescue Committee has estimated that 1,500 people are dying each day as the direct or indirect result of the conflict. It is possible that more families with children had migrated or were living in camps and receiving humanitarian aid for their children. Therefore, these children have better survival chances [13-16].

This study corroborates previous findings in SSA on the effect of mother’s educational attainment on mortality. It clearly shows that higher educational attainment is associated with a lower risk of U5M [17]. However, mother’s educational attainment became statistically insignificant after taking into account other factors such as ongoing conflicts and the general degradation of health services. Obviously, education cannot help in a situation of ongoing conflict when there is no basic public health infrastructure.

Our methods were also able to capture the nonlinear association of the baseline hazard of child’s death. The baseline effects peak at 24, 30 and 36 months. These observed peaks are caused by the large number of deaths reported at these time intervals. Therefore, it is plausible to suspect that this is a “heaping” effect owing to incorrect reporting of a large number of deaths at these ages. Such an assumption reflects digit preference in reporting deaths at 2 and 3 years.

Results of fixed effect factors obtained from our approach are consistent with previous findings that have been reported in literature. This serves as an internal validity check of our approach, confirming that the modelling is able to estimate unobserved factors (e.g., the effect of conflict in the geographic location where the child lives) beyond individual and household factors. For instance, the decreased risk of mortality by age is a well-established demographic fact in SSA [3,4,31-34]. It is likely that the increased build-up of immunity against diseases is one of the reasons survival improves with increasing age. We also observed a rural–urban divide in U5M. The disparity is because of rural children being more likely than their counterparts who live in cities and urban areas to die in the first 5 years of life. The availability and easy access to health care in urban areas could explain the observed disparity by residence.

This study has made policy and methodological contributions above those made by previous studies. In terms of policy, we accounted for spatial factors in child mortality beyond socioeconomic, demographic and health-related determinants in specific contexts. Where the spatial dimension is ignored, population-level socioeconomic variables and health resources cannot sufficiently explain why mortality rates vary across locations. It is well documented that aggregate mortality rates in many developing countries mask spatial variations and that understanding these spatial patterns may lead to the identification of other important determinants of child health. More importantly, war often leads to displacement and concentration of the population in ‘safer’ regions. For this reason, information on spatial patterns in mortality makes an important contribution to programme evaluation and policy development. In terms of methodological shortcomings of past work, we investigated the auto-correlation in the data, non-linear and time varying effects of covariates and the survival nature of mortality data by accounting for the timing of death [3,4].

This study has some limitations. The indicators of mortality presented in this study were calculated from the history of births reported by the women respondents at the time of the survey. As is often the case with retrospective surveys, bias and heaping effects cannot be avoided, in addition to the censoring of the event. Therefore, survival techniques were applied to the data to take into account recall bias, heaping effects and censoring. The other limitation of this study is the potential issue with data quality, because these data were collected in conflict and post-conflict contexts. In conflict situations, it can be difficult to collect reliable data [23,24].

## Conclusions

This study has shown considerable variation in U5M by province in the DRC. U5M remains a very serious public health issue regardless of the ongoing conflict. U5M remains high even in provinces that have not experienced conflict. It is possible that some efforts by the Congolese Government and donor organisations are variable across provinces. This variability is reflected in the overall lack of progress made in reducing the U5MR. Given the observed disparity, it is unlikely that the DRC Government will achieve the MDG 4 on the reduction of U5M by 2015.

## Competing interests

The authors declare that they have no competing interests.

## Authors’ contributions

N-BK conceived of and designed the study; conducted the data analysis and interpretation and was involved in conducting the literature review and drafting the article. PTM was involved in conducting the literature review, interpreting the results and drafting the article. RKM was involved in conducting the literature review, interpreting the results and drafting the article. JBOE was involved in interpreting the results. PDNK was involved in interpreting the results. PKK was involved in interpreting the results. BBK was involved in interpreting the results. All authors performed critical revisions for important intellectual content and read and approved the final manuscript.

## Pre-publication history

The pre-publication history for this paper can be accessed here:

http://www.biomedcentral.com/1471-2458/14/266/prepub

## Supplementary Material

Additional file 1 **Statistical methodology.** Description of Data: We examined the spatial variation in under-five mortality with a flexible Bayesian geo-additive discrete-time survival model. This model has been used and described by the first author of the present study elsewhere in Kazembe et al. [33] and is reproduced in Additional file 1 to facilitate the flow of ideas. Click here for file

## References

[B1] The World Health Report 2005: Make Every Mother and Child Count The World Health Report 2005: Make Every Mother and Child Count 2005 Geneva http://www.who.int/whr/en/10.1080/14034940500217037 16332605

[B2] Nations U The Millennium Development Goals Report 2012 2012 New York

[B3] Mosley WH Chen LC Child survival strategies for research Popul Dev Rev 1984 10 25 45

[B4] Kandala NB Ghilagaber G A geo-additive Bayesian discrete-time survival model and its application to spatial analysis of childhood mortality in Malawi Qual Quant 2006 40 935 957 10.1007/s11135-005-3268-6

[B5] Ministère du plan [République Démocratique du Congo] et and Macro International Inc [Calverton, Marylan, USA) Enquête Démographique et de Santé (EDS-RDC 2007), Rapport Final 2008 Kinshasa, République Démocratique du Congo: Ministère du plan

[B6] Hogan MC Foreman KJ Naghavi M Ahn SY Wang M Makela SM Lopez AD Lozano R Murray CJL Maternal mortality for 181 countries, 1980–2008: a systematic analysis of progress towards Millennium Development Goal 5 Lancet 2010 375 1609 1623 10.1016/S0140-6736(10)60518-1 20382417

[B7] Black RE Global, regional, and national causes of child mortality in 2008: a systematic analysis Lancet 2010 375 1969 1987 10.1016/S0140-6736(10)60549-1 20466419

[B8] Mock N de Buhr E Mukungo M Wemakoy O Public Health Training in the Democratic Republic of Congo: A Case Study of the Kinshasa School of Public Health 2006 Baltimore: John Hopkins Bloomberg School of Public Health

[B9] Ministère de la Santé [République Démocratique du Congo] Etats Généraux de la Santé 1998 Kinshasa, République Démocratique du Congo: Ministère de la Santé

[B10] DRC Ministry of Health and the World Bank Revue des Dépenses Publiques 2007 Kinshasa, République Démocratique du Congo: Ministère de la Santé

[B11] Wembonyama S Mpaka S Tshilolo L Medicine and health in the Democratic Republic of Congo from Independence to the 3rd Republic Med Trop (Mars) 2007 67 447 457 18225727

[B12] World Health Organization Health Action in Crises 2008 Democratic Republic of Congo

[B13] Van Herp M Parqué V Rackley E Ford N Mortality, violence and lack of access to Healthcare in the Democratic Republic of Congo Disasters 2003 27 141 153 doi:10.1111/1467-7717.00225 10.1111/1467-7717.00225 12825437

[B14] Coghlan B Brennan RJ Ngoy P Dofara D Otto B Clements M Stewart T Mortality in the Democratic Republic of Congo: a nationwide survey Lancet 2006 367 44 51 10.1016/S0140-6736(06)67923-3 16399152

[B15] Roberts L Ngoy P Mone C Lubula C Mwezse L Zantop M Despines M Mortality in the Democratic Republic of Congo: Results from a Nationwide Survey 2002 New York: International Rescue Committee (IRC)

[B16] Centers for Disease Control and Prevention (CDC) Elevated mortality associated with armed conflict—Democratic Republic of Congo, 2002 MMWR Morb Mortal Wkly Rep 2003 52 469 471 12807080

[B17] Kandala N-B Emina JBO Nzita PD Cappuccio FP Diarrhoea, acute respiratory infection, and fever among children in the Democratic Republic of Congo Soc Sci Med 2009 68 1728 1736 10.1016/j.socscimed.2009.02.004 19285371

[B18] UNICEF DRC Pauvreté des Enfants et Disparités en République Démocratique du Congo 2008 Kinshasa

[B19] UNICEF The State of the World’s Children, 2010: UNICEF Annual Report 2010 New York

[B20] Doctor without Borders Food, Nutrition and Mortality Situation of IDP’s in Dubie, Katanga 23–25 March 2006 http://www.msf.org.uk/sites/uk/files/2008_Starved_for_Attention__Wake_Up_to_the_Crisis_of_Malnutrition_200907240853.pdf

[B21] United Nations Development Programme (UNDP) United Nations Human Development Report 2011: Sustainability and Equity: A Better Future for All 2011 New York

[B22] The World Bank World Development Indicators 2012 2012 Washington DC: The World Bank

[B23] République du Zaïre/UNICEF, PNUD, OMS Enquête sur la Situation des Enfants et des Femmes (Ensef) 1995 Kinshasa: MICS1

[B24] RDC/UNICEF Enquête Nationale sur la Situation des Enfants et des Femmes MICS2, Rapport d’Analyse 2002 Kinshasa

[B25] African Development Bank The Democratic Republic of Congo: 2013–2017 Country Strategy Paper 2013 Regional Department Centre (ORCE/CDFO) [http://www.afdb.org/fileadmin/uploads/afdb/Documents/Project-and-Operations/Democratic%20Republic%20of%20Congo%20-%202013-2017%20-%20Country%20Strategy%20Paper.pdf]

[B26] Hennerfeind A Brezger A Fahrmeir L Geoadditive survival models J Am Stat Assoc 2006 101 1065 1075 10.1198/016214506000000348

[B27] Fahrmeir L Lang S Bayesian inference for generalized additive mixed models based on Markov random field priors Appl Stat Series C 2001 50 11 30

[B28] Spiegelhalter DJ Best NG Carlin BP van der Linde A Bayesian measures of model complexity and fit (with discussion) J R Stat Soc B 2002 64 1 34 10.1111/1467-9868.02022

[B29] Brezger A Kneib T Lang S BayesX-Software for Bayesian inference based on Markov chain Monte Carlo simulation techniques J Stat Softw 2005 14 11

[B30] Brooks SP Gelman A General methods for monitoring convergence of iterative simulations J Comput Graph Stat 1998 7 434 455

[B31] Bolstad WM Manda SO Investigating child mortality in Malawi using family and community random effects: a Bayesian analysis J Am Stat Assoc 2001 96 12 19 10.1198/016214501750332659

[B32] Kazembe L Mpeketula PMG Quantifying spatial disparities in neonatal mortality using a structured additive regression model PLoS One 2010 5 e11180 10.1371/journal.pone.0011180 20567519 PMC2887370

[B33] Kazembe L Clarke A Kandala N-B Childhood mortality in Sub-Saharan Africa: insight of small-scale geographical inequalities from census data BMJ Open 2012 2 e001421 doi:10.1136/bmjopen-2012-001421 23089207 10.1136/bmjopen-2012-001421 PMC3488715

[B34] Akoto EM Mortalité Infantile et Juvénile en Afrique: Niveaux et Caractéristiques, Causes et Déterminants 1985 Louvain-la-Neuve: CIACO

