# Supplementary material for: Child mortality in the Democratic Republic of Congo: cross-sectional evidence of the effect of geographic location and prolonged conflict from a national household survey
Source: BMC Public Health. 2014 Mar 20;14:266. doi: 10.1186/1471-2458-14-266 (PMC4234186; doi:10.1186/1471-2458-14-266)
Supplement: Additional file 1 — Statistical methodology. Description of Data: We examined the spatial variation in under-five mortality with a flexible Bayesian geo-additive discrete-time survival model. This model has been used and described by the first author of the present study elsewhere in Kazembe et al. [33] and is reproduced in Additional file 1 to facilitate the flow of ideas. [file 1471-2458-14-266-S1.docx]

**Additional file 1. Statistical Methodology**

We examined the spatial variation in under-five mortality with a flexible Bayesian geo-additive discrete-time survival model. This model has been used and described by the first author of the present study elsewhere in Kazembe et al. 2012 (http://creativecommons.org/licenses/by-nc/2.0/) and is reproduced below (from “Model Specification” to the end of the additional file), with some textual changes, to facilitate the flow of ideas [1]. This models mortality events as person-specific Cox processes while controlling spatial dependence and possibly nonlinear effects of covariates within a simultaneous and coherent regression framework.

As suggested in Hennerfeind et al. [2], this modelling framework captures a wide range of issues, including individual risk of early childhood mortality affecting spatial frailties. We used a Bayesian geo-additive discrete-time survival model instead of the general spatial Cox regression model. We applied a full Bayesian approach based on Markov priors using Markov Chain Monte Carlo (MCMC) techniques for inference and model checking [3]. For model choice, we used the Deviance Information Criterion (DIC), developed as a measure of fit and model complexity [4].

**Model specification**

In studying the survival of the child, we assume *T* as time to event or survival time with *t* as the actual realization. The probability that a survival time *T* is less than or equal to some value *t* is given by . In our context, *F*(*t*) is the cumulative probability that a child dies at or before some given time *t*, so *F*(*t*) = *P*[Child dies at time ≤ *t*]. The instantaneous probability that an event will occur at any given small interval is defined as *F*'(*t*) = *f*(*t*). The proportion of children surviving to time *t* or beyond is derived as *S*(*t*) = 1−*F*(*t*), which is also known as the survivor function. An important approach is to consider the duration analysis through the hazard rate. The hazard rate, which links the survival and failure functions, is of the form , equivalent to *h*(*t*)=−*logS*'(*t*). The hazard rate, unlike the survivor function, describes the risk or event of “failure” given that the individual has survived up to point *t*.

In the analysis of child mortality, our interest is to answer this question: Given that the child has survived up to month *t*, what is the likelihood he/she will survive in the subsequent months? Additionally, we are interested in how the hazard rate varies with respect to certain covariates. For instance, will the hazard be the same for children living in urban and rural areas? One way to analyse such data is to use Kaplan-Meier survival curves and the log-rank test. This is an exploratory analysis that permits assessment of any differences in child survival by various covariates.

An alternative which captures the effect of covariates is to use Cox regression models, commonly referred to as proportional hazards models (PHM). It should be pointed out, however, that various statistical models may be constructed, see Box-Steffensmeier and Jones (2004) for an overview on the topic [5]. Several issues that are not part of the analysis of other types of data must be considered when analyzing survival data. Central issues are the censoring and truncation of survival data, the existence of time-varying covariates, the occurrence of multiple causes of death, the question of whether occurrences of events were recorded in discrete-time and the possibility of group-risk factors and confounders acting on the hazard. Thus, a more general model that incorporates all of these issues if they are present in the data is needed.

We propose using a more general Cox model that captures a wide range of issues, including spatial frailties. Thus, a spatial Cox regression model was applied to determine the factors associated with the risk of early childhood mortality [6]. Assume that is the observed number of months lived or the censoring time for *j*-th child in area *i*. Under Cox’s model, the hazard function at time *T* = *t* is given by

(1)

where is the baseline hazard at time *t*, and the βs are a vector of regression coefficients for the fixed and time-invariant variables (. The exponent of a coefficient, exp(β), is interpreted as the hazard ratio (HR), that is, the ratio of instantaneous risks, which is assumed to be constant over time. The HR compares rates of deaths in one group to those of some reference group for a categorical variable, and to the mean for a continuous variable.

Since individuals are clustered in geographical regions, a group-specific random frailty term, , was introduced to augment the Cox model:

(2)

The above model indicated that childhood survival was influenced by both individual-specific factors () and group-specific environmental factors (). Here it was assumed that the environmental factors were approximated by geographical locations. In the case of geographical regions, spatially distributed random effects were assumed, while for the other unstructured heterogeneity, a random effect, , was specified such that . Fitting model (2) assumed a semiparametric additive predictor, which is known as geoadditive survival model [2],

(3)

where is the log-additive predictor at time *t* for child *j* in area *i*. The term is the log baseline hazard effect at time *t*. The other terms are as defined above.

### Estimation: fully Bayesian approach

#### Prior distributions for covariate effects

Modelling and inference use the fully Bayesian approach. In the Bayesian formulation, the specification of the proposed model (Equation 3) is completed by assigning priors to all unknown parameters. For the fixed regression parameters, a suitable choice is the diffuse prior (i.e. *p*(γ)∝*const*), but a weakly informative Gaussian prior is also possible. The baseline hazard effect, , was assigned a penalized spline with a second order random walk prior. Similarly, the time and continuous covariates were estimated non-parametrically through smoothness priors. We use the second-order Gaussian random walk prior to allow enough flexibility while penalising abrupt changes in the function, as suggested by Brezger et al. [7]. The prior can be expressed in the pairwise difference form as

(4)

where and is the variance, with diffuse priors , for initial values.

For the unstructured spatial heterogeneity term, is assumed to follow an exchangeable Gaussian prior with zero mean and variance, , i.e., . Finally, for the spatial components , we assign a Markov random field (MRF) prior [8]. This is analogous to random walk models. The conditional distribution of , given adjacent areas , is a univariate normal distribution with mean equal to the average values of ’s neighbouring areas and variance equal to divided by the number of adjacent areas. This leads to a joint density of the form

(5)

where *i*~*j* denotes that area *i* is adjacent to *j*, and assumes that parameter values and in adjacent areas are similar. The degree of similarity is determined by the unknown precision parameter .

By writing , , and , for a well defined design matrix *Z* and a (possibly high-dimensional) vector of regression parameters β, all different priors (Equations 4–5) can be expressed in a general Gaussian form

(6)

with an appropriate penalty matrix . Its structure depends on the covariate and smoothness of the function. In most cases, is rank deficient and hence the prior for is improper. For the variances we assume inverse Gamma priors , with hyperparameters , chosen such that this prior is weakly informative.

#### Posterior distribution

Fully Bayesian inference is based on the analysis of posterior distribution of the model parameters. In general, the posterior is highly dimensional and analytically intractable, which makes direct inference almost impossible. This problem is circumvented by using MCMC simulation techniques, whereby samples are drawn from the full conditional of parameters given the rest of the data. Under conditional independence assumptions, the posterior distribution for the Bernoulli model is given by Bayes’ Theorem

$=L(data|b,t^{2},g)\times\left\{\prod_{i=1}^{p} p(\beta_{j}|\tau_{j}^{2})p(\tau_{j}^{2})\right\}p(\gamma)$ (7)

where the quantity is the prior density function, and denotes the likelihood of the data. More specifically, the posterior is given by

(8)

where is a binary indicator coded 1 if an event occurs and 0 if an event does not occur at time *t*. For updating the full conditionals of parameters, we use a hybrid MCMC sampling scheme of the iteratively weighted least squares (IWLS) proposals, developed for generalised linear mixed models by Brezger [7], and the Metropolis-Hastings algorithm. Full details are presented elsewhere [7, 9].

### Data Analysis

A number of models were explored. The first model (*M*0) explored unstructured variation in child *i* at provincial level *k*.

*M*0:

The second set of models estimated fixed effects only (*M*1*a*), and then we adjusted for unstructured random effects at province level (*M*1*b*).

*M*1*a*:

*M*1*b*:

The last set of models combined fixed and random effects at province level. In model *M*3*a*, we estimated structured spatial effects at province level and unstructured effects at province level, and model (*M*3*b*) improved model *M*3*a* by incorporating fixed effects.

Model comparison was based on the DIC [4]. This is given by , where is the deviance of the model evaluated at the posterior mean of the parameters and represents the fit of the model to the data. The component is the effective number of parameters, which assesses the complexity of the model. Because small values of indicate a good fit and small values of indicate a parsimonious model, small values of DIC indicate a better model. Models with differences in DIC of < 3 compared with the best model cannot be distinguished, whereas those ranging from 3 to 7 can be weakly differentiated [4].

### References

1. Kazembe L, Clarke A, Kandala N-B: **Childhood mortality in Sub-Saharan Africa: insight of small-scale geographical inequalities from census data.** *BMJ Open* 2012, **2**:e001421. doi:10.1136/bmjopen-2012-001421.
2. Hennerfeind A, Brezger A, Fahrmeir L: **Geoadditive survival models.** *J Am Stat Assoc* 2006, **101**:1065-1075.
3. Fahrmeir L, Lang S: **Bayesian inference for generalized additive mixed models based on Markov random field priors.** *Appl Stat Series C* 2001, **50**:11-30.
4. Spiegelhalter DJ, Best NG, Carlin BP, van der Linde A: **Bayesian measures of model complexity and fit (with discussion)**. *J R Stat Soc Series B Stat Methodol* 2002, **64**:1-34.
5. Box-Steffensmeier JM, Jones BS: *Event History Modeling: A Guide for Social Scien­tists.* New York: Cambridge University Press; 2004.
6. Cox DR: **Regression models and life-tables.** *J R Stat Soc Series B Stat Methodol* 1972, **34**:187-220.
7. Brezger A, Kneib T, Lang S: **BayesX: Software for Bayesian inference based on Markov chain Monte Carlo simulation techniques.** *J Stat Softw* 2005, **14**:11.
8. Brooks SP, Gelman A: **General methods for monitoring convergence of iterative simula­tions**. *J Comp Graph Statist* 1998, **7**:434-455.
9. Carlin BP, Banerjee S, Wall M: **Frailty modelling for spatially correlated survival data with application to infant mortality in Minnesota.** *Biostatistics* 2003, **4**:123-142.
